# Antisecretory factor for treatment of peritumoral edema in glioblastoma patients

**DOI:** 10.1007/s00701-025-06481-z

**Published:** 2025-03-08

**Authors:** Louise Carstam, Tomás Gómez Vecchio, Monika Lyczak, Hanna Åberg, Asgeir S. Jakola, Eva Jennische, Stefan Lange, Kliment Gatzinsky

**Affiliations:** 1 Department of Neurosurgery, Sahlgrenska University Hospital, 41345 Gothenburg, Sweden; 2 Department of Clinical Neuroscience, Institute of Neuroscience and Physiology, Sahlgrenska Academy, University of Gothenburg, 405 30 Gothenburg, Sweden; 3 Department of Neurology, Skaraborg Hospital, Skövde, Sweden; 4 Department of Neurology, Northern Älvsborg County Hospital, Trollhättan, Sweden; 5 Department of Medical Biochemistry and Cell Biology, Institute of Biomedicine, Sahlgrenska Academy, University of Gothenburg, 405 30 Gothenburg, Sweden; 6 Department of Clinical Microbiology, Sahlgrenska University Hospital, 41345 Gothenburg, Sweden; 7 Department of Infectious Diseases, Institute of Biomedicine, University of Gothenburg, 405 30 Gothenburg, Sweden

**Keywords:** Glioblastoma, Antisecretory factor, Salovum, Peritumoral edema

## Abstract

**Purpose:**

Glioblastoma (GBM) is an aggressive brain tumor often accompanied by a vasogenic peritumoral edema, which contributes to symptoms both at diagnosis and during later stages of the disease. Previous studies have suggested effectiveness of the endogenous protein, Antisecretory Factor (AF), in reducing the intracranial pressure in cytotoxic brain edema after trauma. Interestingly, AF also seems to carry antineoplastic effects in experimental GBM models. This study investigated whether AF reduces peritumoral edema in GBM patients. As a secondary aim, we assessed potential effects on tumor progression by AF.

**Methods:**

Fifteen newly diagnosed GBM patients were treated for 7 days preoperatively with AF in addition to standard of care (SOC) treatment with corticosteroids. The change in edema volume was assessed volumetrically using T2/FLAIR weighted MRI and compared to a control group of 10 GBM patients receiving SOC only.

**Results:**

At baseline the mean tumor volume for the entire cohort was 35.7 cm3 with a mean edema of 62.2 cm3. There was no significant difference in edema volume change between the AF treated patients, who demonstrated a mean edema reduction of 7.1cm3 (95%CI -5.4–19.6), and the controls, 11.3cm3 (95%CI -0.8–23.5), *p* = 0.61. No difference was observed in tumor volume change between the two groups, *p* = 0.79. No adverse treatment effects were noted.

**Conclusion:**

Treatment with AF in addition to SOC does not seem to reduce the peritumoral edema in GBM patients. The treatment was well tolerated. The lack of edema-reducing effect may be related to the different pathophysiological properties of vasogenic and cytotoxic edema.

## Introduction

Glioblastoma (GBM) is the most common malignant primary brain tumor in adults [23]. There is currently no cure for the disease, and the median survival despite full multi-modal treatment is around 15–16 months, with a 5-year survival rate of less than 3% [24, 34]. The tumor is usually surrounded by an extensive edema that typically causes or aggravates the symptoms from the disease, including neurological or neurocognitive decline and increased intracranial pressure (ICP) [26]. Currently glucocorticoids (Dexamethasone/Betamethasone) are widely used as standard of care (SOC) to reduce the edema and its effects. However, albeit effective in edema reduction, glucocorticoids also entail well-known side-effects from the endocrine, musculoskeletal, neuropsychiatric, and immunologic systems inter alia [3, 27]. In addition, there are concerning reports of possible adverse effect on survival in GBM patients from the use of glucocorticoids [15, 29, 36]. Alternative agents for reducing peritumoral brain edema would therefor be beneficial.

Antisecretory Factor (AF) is an endogenous protein that displays antisecretory and anti-inflammatory properties in higher vertebrates and humans [22]. The most evaluated effects of AF have been observed from the gastro-intestinal tract with a protective effect against diarrheal disease and intestinal inflammation [10, 21, 37, 38]. The protein has also been reported to cross the blood brain barrier [18] and evidence for beneficial effect on elevated ICP and possibly brain edema in animals and humans is mounting [2, 6, 7, 13, 14]. Further, it has been shown in animal experiments that exogenous administration of AF reduces the intratumoral fluid pressure (IFP), and that AF may induce antitumor activity in murine GBM models, preventing tumor growth [1, 16, 19, 28].

For human administration of AF, a medical food classified by the European Union, Salovum® (freeze-dried egg yolk powder produced from hens fed with AF-enriched cereals), has been used in various earlier studies including one recent study of eight patients with GBM [8–10, 20, 37, 38]. In the present study, the aim was to investigate any signals of edema reduction following peroral administration of Salovum in patients with GBM and peritumoral edema. Further, as a secondary aim we wanted to compare tumor growth in patients receiving AF, compared to that of patients treated with SOC only. Finally, we aimed to assess safety and tolerability of oral AF treatment in this group of patients.

## Materials and methods

### Patient population and intervention

From the weekly regional brain tumor multidisciplinary team (MDT)-conference, adult patients with radiological findings characteristic of GBM demonstrating significant peritumoral edema were included.

Fifteen patients were treated with orally administered AF in the form of Salovum (16 g three times daily, for a total of 48 g per day, dissolved in a glass of water or juice) for seven days in addition to SOC with corticosteroids. MRI scans were performed immediately before initiation and just after termination of the treatment. Ten control patients who received SOC only, followed the same MRI scan protocol of two scans with a seven day interval. In both groups, the second scan was typically coinciding with the scheduled pre-operative imaging. Betamethasone was administered as part of SOC in both groups.

### Safety and adverse events

All treated patients were assessed for tolerability, adverse events, and compliance with the regimen at the end of treatment.

### Volumetric analysis

**Fig. 1:**
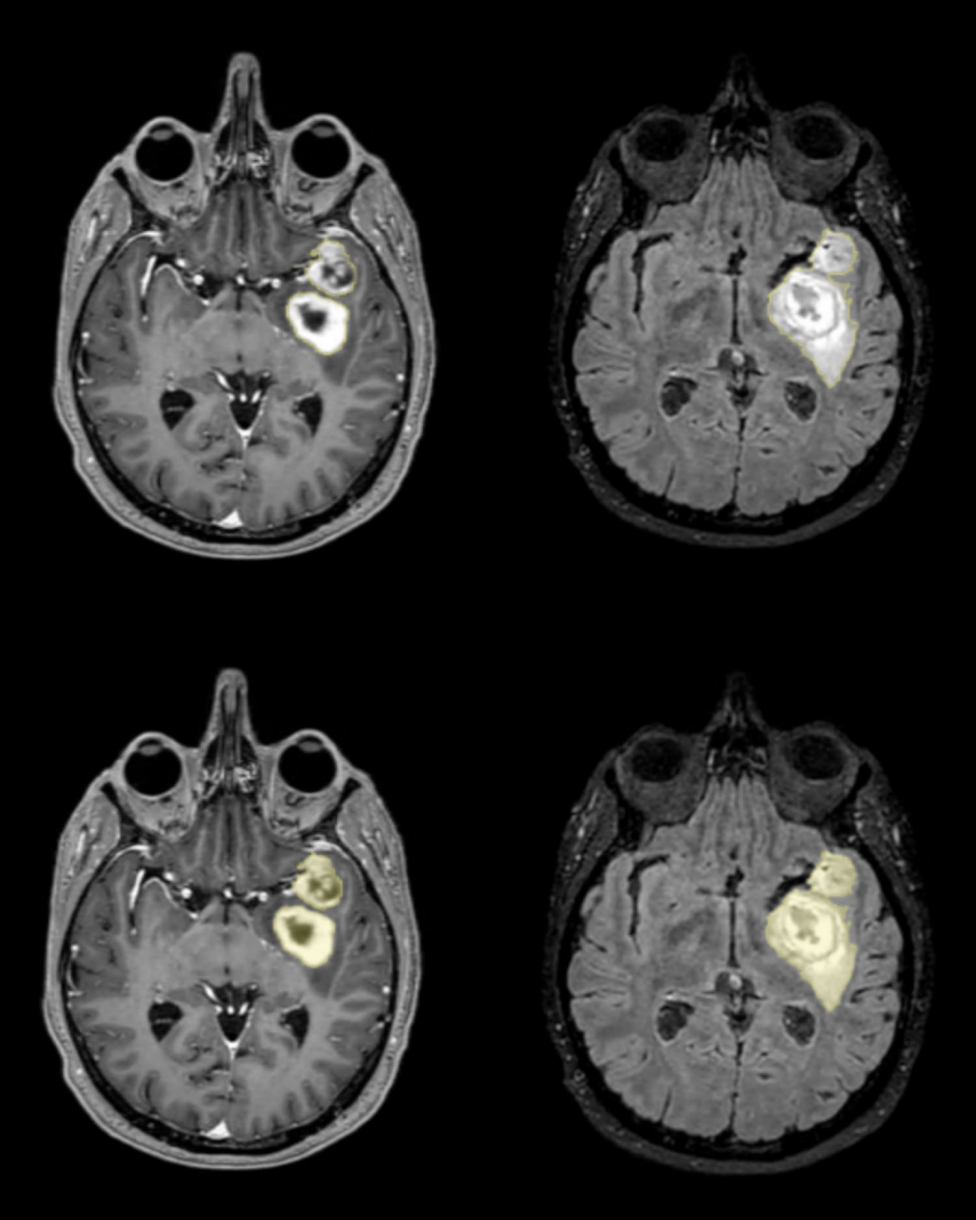
The outlines (top) and color overlap (bottom) of a GBM on a T1Gd slice are shown to the left. The images to the right are corresponding outline (top) and color overlap (bottom) of the peritumoral edema from the same tumor on a FLAIR weighted MRI slice. The delineations were performed with the semi-automatic software “3DSlicer” T1Gd = T1-weighted, Gadolinium contrast enhanced MRI. FLAIR = Fluid Attenuated Inversion Recovery sequenced MRI

Tumor and edema volumes were quantified through semi-automatic region of interest (ROI)-delineation performed with the open-source software “3DSlicer” version 4.6.2 [12]. The segmentation was performed by trained personnel using the “3DSlicer” software, (TGV), and in all cases verified by a senior neurosurgeon (LC). The scans were investigated in a randomized order and the patient id and the dates of the MRI scans were blinded to the assessors, so that it was unknown whether the images belonged to treated patients or to controls and whether a scan was from before or after AF treatment. The tumor volumes were measured on T1 weighted images with Gadolinium contrast (T1Gd) with thin slices (0.5–1.0 mm), whereas the peritumoral edema was assessed from T2/FLAIR volumes with equally thin slices. Figure 1 shows MRI images of a tumor with peritumoral edema.

### Statistics

All analyses were performed with SPSS, version 29 (Chicago, IL, USA). Statistical significance level was set to *p* < 0.05. All tests were two-sided. Central tendencies are presented as means ± Standard deviation (SD) or with 95% Confidence Intervals (CI), or as median with first and third quartile (Q1; Q3) if skewed. Volume differences over time (between first and second MRI scan) between groups (treated vs non-treated) were compared with repeated measures ANOVA.

## Results

### Baseline data and demographics

Baseline values for AF treated patients and controls are presented in Table 1.

**Table 1:** Baseline metrics for AF treated subjects and controls

Variable	All patients *N* = 24	AF treated *N *= 14	Controls *N* = 10	*p*-value (AF vs controls)
Age, mean (SD)	64.0 (10.8)	63.8 (12.0)	64.2 (9.3)	0.94
Sex, Female (%)	5 (20.8)	4 (28.6)	1 (10)	0.36
Mean tumor volume in cm3 (SD)	35.7 (22.8)	36.7 (23.4)	34.3 (23.1)	0.80
Mean Edema* volume in cm3 (SD)	62.2 (38.4)	73.3 (37.1)	46.8 (36.6)	0.10
Median number of days on steroids prior to first study MRI (Q1; Q3)	10 (5.8;13.0) missing 2	10 (7; 13.5) missing 1	9 (5.0; 12.5) missing 1	0.44
Median steroid dosage in mg/day during time between study examinations (Q1; Q3)	2 (2; 4) missing 2	2 (2; 2.15) missing 1	2 (2; 4.4) missing 1	0.46

*The Edema Volume was calculated from the FLAIR/T2 image by subtracting the Tumor Volume on T1Gd

For technical reasons one treated patient was excluded from analysis. This was due to MRI scan not adhering to protocol with a non-volumetric scan, applying thicker slices (5mm) and interslice gap, leaving 14 treated patients and 10 control patients for analysis.

There were no statistically significant differences between controls and treated patients at baseline. However, a tendency for larger edema volumes were seen in the AF treatment group.

### Edema reduction and tumor progression

**Fig. 2:**
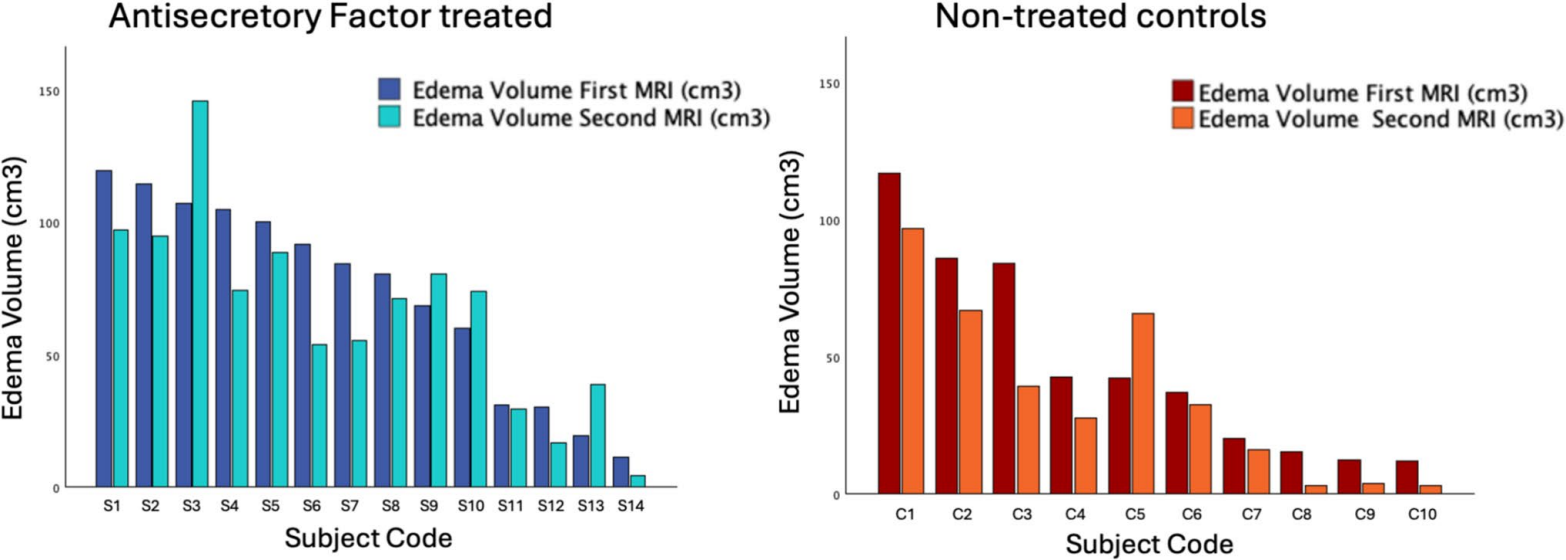
Changes in edema volume over the seven-day interval between the first and second MRI scans are shown for each individual in the AF-treated group (left) and the non-treated control group (right)

There was a reduction in edema volume between the first MRI-scan (before AF treatment) and at second scan (7 days later), for the entire cohort with a mean edema reduction of 8.9 cm3, (95%CI: 0.6–17.1), *p* = 0.04, for details see Table 2. There was, however, no significant difference in tumor edema reduction between the two groups (AF treated vs control patients) (*p* = 0.61). Group level numeric and percentage difference in edema and tumor volume are presented in Table 2 whereas edema changes per individual are shown in Fig. 2.

**Table 2:** Edema and tumor volumes after treatment period

Variable	All patients *N* = 24	AF treated *N* = 14	Controls *N* = 10	p-value (AF vs controls)
Mean Edema reduction, cm3 (SD)	8.9 (10.6)	7.1 (21.6)	11.3 (17.0)	0.61
Mean Edema reduction in percentage (SD)	17.8% (41.7)	7.2% (40.8)	32.8% (40.2)	0.14
Mean tumor volume increase, cm3 (SD)	1.8 (4.4)	2.0 (3.7)	1.5 (5.4)	0.79
Mean tumor volume increase in percentage (SD)	8.9% (20.5)	6.2% (8.5)	12.8% (30.8)	0.44

**Fig. 3:**
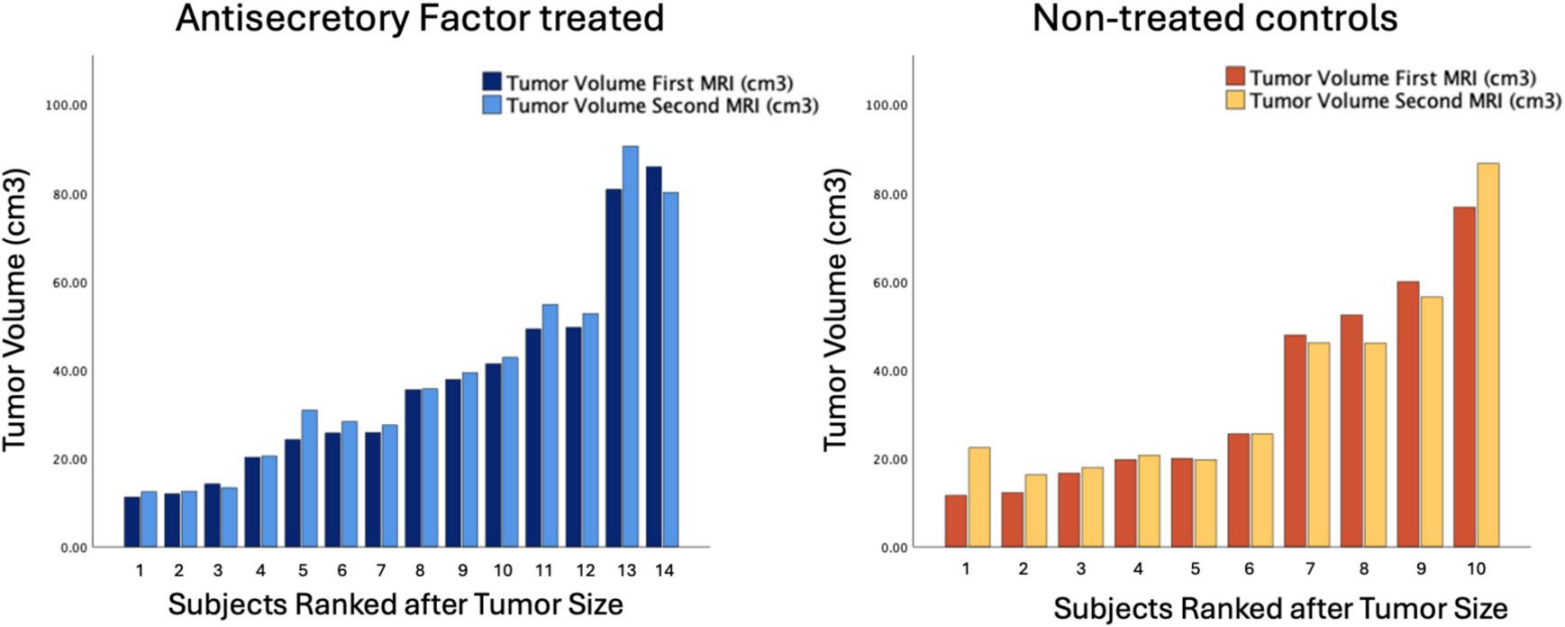
Changes in tumor volume over the seven-day interval between the first and second MRI scans are shown for each individual in the AF-treated group (left) and the non-treated control group (right)

For all 24 GBM patients, a tendency for tumor progression was seen between the first and second scan on group level, with a mean tumor volume increase of 1.8 cm3 (95%CI: 0.0–3.6), *p* = 0.07. There was no significant difference pertaining to tumor size increase between AF treated patients and controls (*p* = 0.79). Tumor size change per individual is visualized in Fig. 3.

### Feasibility and safety

All treated patients were able to complete the treatment, which was well tolerated, and no adverse events were recorded.

## Discussion

The addition of AF to SOC did not affect the peritumoral edema in newly diagnosed GBM patients in this pilot study. Neither did we see any signals of reduced tumor volume. The AF treatment appears to be safe and well tolerated, as all treated subjects adhered to the regimen without any adverse events.

Two previous clinical studies have suggested a favorable effect of AF on brain edema induced by trauma [6, 13]. Orally, intravenously or intranasally administered AF has also been shown to decrease the ICP in murine models with trauma-related ICP increase [17]. By contrast, in a study investigating the potential ICP-reducing effects of AF in idiopatic normal pressure hydrocephalus and idiopathic intracranial hypertension, no such effect was observed[9]. These observations indicate that AF is not effective in all types of intracranial fluid disturbances.

Cerebral edema generally falls into two main types, referred to as cytotoxic or vasogenic edema, where the former is mainly intracellular and may be caused by for example trauma or hypoxia, while the latter involves fluid accumulation in the extracellular space and typically is seen surrounding brain tumors [25, 32]. While corticosteroids are effective and widely used in treating peritumoral vasogenic edema, they are normally not beneficial for alleviating trauma-induced brain swelling or other forms of cytotoxic cerebral edema [30]. Conversely, the promising effect from AF in traumatic edema, may not be translatable to the vasogenic tumor edemas.

The exact mechanisms of action of AF are still not known but certain types of membrane bound GABA receptors seem to be involved in binding the active AF-16 peptide to the cell in order to exert its effect at the cellular level in the central nervous system (CNS) where it theoretically may counterbalance the glutamatergic excitotoxicity found in cytotoxic (but not in vasogenic) edemas [4, 5, 18, 31–33]. In this context, it has been suggested that AF may exert its influence on brain cells through uptake via endocytosis into the cells with regulation of autophagy activity, restitution of mitochondrial dynamics, and cell membrane healing with normalized intra-extracellular Na + transportation, thereby reducing intracellular damage and fluid build up with cell swelling caused by traumatic brain injury [35]. This type of AF-mediated direct influence on brain cell function, which mainly involves astrocytes, may not apply to vasogenic edema and endothelial cells where the intravascular Na + outflow due to damage of the blood–brain barrier results in extracellular fluid accumulation in the cerebral parenchyma which typically evokes an increase of brain volume and ICP [17, 25, 32, 35].

In the present study, consideration must be given to whether the doses and treatment duration were adequate for AF to achieve its intended effect to reduce brain edema. The dosage used was based on previously applied regimens in studies of patients suffering from inflammatory diseases of various origins as well as extrapolation from one study of patients with trauma induced brain edema [10, 11, 13, 21, 37, 38]. However, higher doses than in the present study have been used in a recent small study in GBM patients assessing survival [8].

Another important point to consider is the simultaneous administration of corticosteroids which is part of SOC for newly diagnosed GBM with edema. This treatment may have obscured potential effects of AF. Accordingly, it would have been ideal to administer the treatment to patients without prior or ongoing steroid use. However, we found this difficult from an ethical standpoint, as many GBM patients are in urgent need of corticosteroids at the time of diagnosis.

## Summary

This pilot study demonstrates that AF treatment is feasible and well tolerated in newly diagnosed GBM patients with peritumoral edema but that no effects were observed beyond those of corticosteroids. There are plausible physiological explanations to this lack of response related to the nature of different types of brain edemas and the current knowledge of AFs mechanisms of action. For future research, a subgroup of patients who exhibit radiological edema, but are not in urgent need for edema alleviation, could potentially be considered for upfront treatment with AF instead of, or compared to, steroids. Further, a dose response investigation for optimal Salovum dosage would ideally be performed prior to such a study.

### Strengths and limitations

This study was conducted as a pilot and feasibility study with adjoining limitations such as the small sample size and the simultaneous Betamethasone treatment, as well as an uncertainty regarding the optimal Salovum dosage in relation to the intended effect in patients with GBM. Strengths of the study include its prospective, controlled and blinded (for all image interpretation) nature.

## Conclusion

AF treatment of newly diagnosed GBM patients proved feasible but did not reduce vasogenic peritumoral edema beyond the effect of corticosteroids. This lack of edema reducing effect, compared to the positive effect of AF that has been suggested in earlier studies regarding reduction of traumatically induced brain edema, may depend on the different molecular and pathophysiological properties of vasogenic and cytotoxic edema.

## Data Availability

The data that support the findings of the present study are not publicly available, due to them containing information that could compromise research participant privacy/consent. The data are however available upon reasonable request to the authors.
